# Crystal structure and Hirshfeld surface analysis of hexa­kis­(μ-benzoato-κ^2^ *O*:*O*′)bis­(pyridine-3-carbo­nitrile-κ*N* ^1^)trizinc(II)

**DOI:** 10.1107/S2056989017016899

**Published:** 2017-11-28

**Authors:** Tuncer Hökelek, Elif Özbek, Mustafa Sertçelik, Çiğdem Şahin Yenice, Hacali Necefoğlu

**Affiliations:** a Department of Physics, Hacettepe University, 06800 Beytepe, Ankara, Turkey; b Department of Chemical Engineering, Kafkas University, 36100 Kars, Turkey; c Department of Chemistry, Kafkas University, 36100 Kars, Turkey; d International Scientific Research Centre, Baku State University, 1148 Baku, Azerbaijan

**Keywords:** crystal structure, zinc(II), transition metal complexes of benzoic acid derivatives

## Abstract

The asymmetric unit of the title complex, [Zn_3_(C_7_H_5_O_2_)_6_(C_6_H_4_N_2_)_2_], contains one half of the complex mol­ecule, *i.e.* one and a half Zn^II^ cations, three benzoate (Bnz) and one pyridine-3-carbo­nitrile (Cpy) mol­ecule; the Bnz anions act as bidentate ligands through the carboxyl­ate O atoms, while the Cpy ligand acts as a monodentate N(pyridine)-bonding ligand. The complete centrosymmetric trinuclear complex thus comprises a linear array of three Zn^II^ cations. The central Zn^II^ cation shows an octa­hedral coordination and is bridged to each of the terminal Zn^II^ cations by three Bnz anions. By additional coordination of the CPy ligand, the terminal Zn^II^ cations adopt a trigonal–pyramidal coordination environment. In the crystal, the Bnz anions link to the Cpy N atoms *via* weak C—H⋯N hydrogen bonds, forming a two-dimensional network. C—H⋯π and π–π inter­actions [between the benzene and pyridine rings of adjacent mol­ecules with an inter­centroid distance of 3.850 (4) Å] help to consolidate a three-dimensional architecture. The Hirshfeld surface analysis confirms the role of H-atom contacts in establishing the packing.

## Abstract 2

The asymmetric unit of the title complex contains one half of the complex mol­ecule, one and a half Zn^II^ cations, three benzoate (Bnz) and one pyridine-3-carbo­nitrile (CPy) mol­ecule; the Bnz anions act as bidentate ligands through the carboxyl­ate O atoms, while the Cpy anion acts as a monodentate N(pyridine)-bonding ligand. The complete centrosymmetric trinuclear complex thus comprises a linear array of three Zn^II^ cations. In the crystal, the Bnz anions link to the Cpy N atoms *via* weak C—H⋯N hydrogen bonds, forming a two-dimensional network. The Hirshfeld surface analysis confirms the role of H-atom contacts in establishing the packing.

## Chemical context   

The structure–function–coordination relationships of the aryl­carboxyl­ate ion in Zn^II^ complexes of benzoic acid deriv­atives change depending on the nature and position of the substituent groups on the benzene ring, the nature of the additional ligand mol­ecule or solvent, and the pH and temperature of synthesis (Shnulin *et al.*, 1981 ▸). When pyridine and its derivatives are used instead of water mol­ecules, the structure is completely different (Catterick *et al.*, 1974 ▸). The solid-state structures of anhydrous Zinc(II) carboxyl­ates include one-dimensional, two-dimensional and three-dimensional polymeric motifs of different types, while discrete monomeric complexes with octa­hedral or tetra­hedral coordin­ation geometry are found if water or other donor mol­ecules are coordinated to Zn (Usubaliev *et al.*, 1992 ▸). The structure determination of the title compound, (I), a trinuclear zinc complex with six benzoate anions and two neutral pyridine-3-carbo­nitrile ligands, was undertaken in order to compare the results obtained with those reported previously. In this context, we synthesized the Zn^II^-containing title compound, hexa­(μ-benzoato-*κ^2^O*,*O′*)bis­(pyridine-3-carbo­nitrile-*κN*)trizinc(II), [Zn_3_(C_7_H_5_O_2_)_6_(C_6_H_4_N_2_)_2_], and report herein its crystal and mol­ecular structures as well as a Hirshfeld surface analysis.
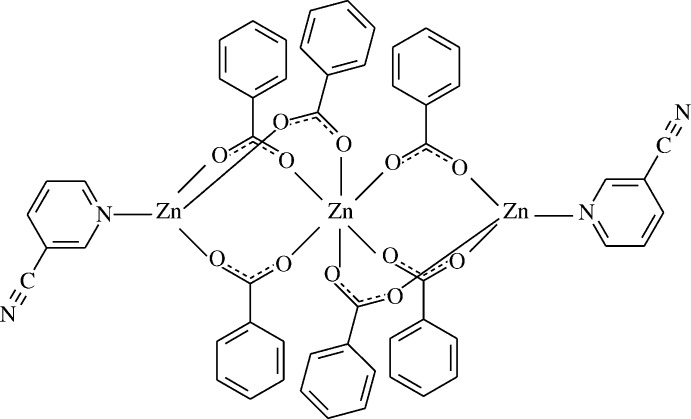


## Structural commentary   

**Figure 1:**
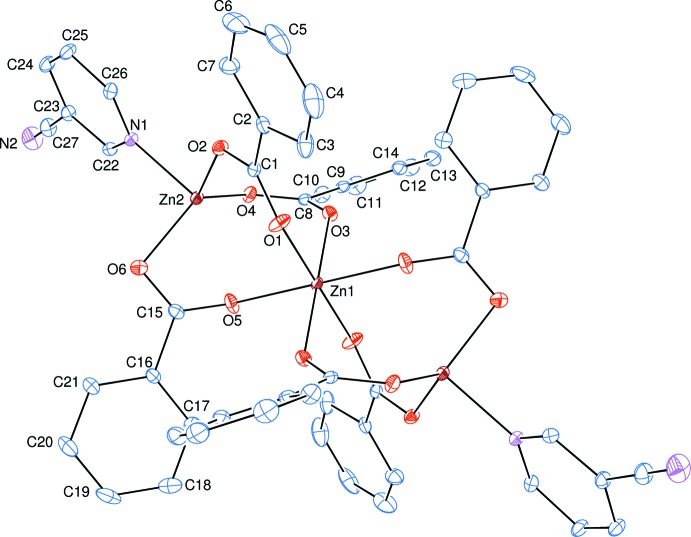
The mol­ecular structure of the title complex with the atom-numbering scheme. Unlabelled atoms are related to labelled ones by the symmetry operation (1 − *x*, 2 − *y*, 1 − *z*). Displacement ellipsoids are drawn at the 30% probability level. H atoms have been omitted for clarity.

The mol­ecular structure of the title complex (I) is formed by a centrosymmetric array of three Zn^II^ cations, which are coord­in­ated by six benzoate anions and two neutral pyridine-3-carbo­nitrile ligands. The middle Zn^II^ cation occupies a special position and lies on a crystallographic inversion centre. The benzoate anions act as bidentate ligands, bridging two pairs of Zn^II^ cations. The pyridine-3-carbo­nitrile ligands are monodentately coordinated through the pyridine N atoms (Fig. 1 ▸).

In the title complex, (I), the four carboxyl­ate O atoms (O1, O3, O1^i^ and O3^i^) of the two symmetry-related, bidentately coordinated benzoate anions around the Zn1 atom form a slightly distorted square-planar arrangement, while the slightly distorted octa­hedral coordination sphere is completed by the two carboxyl­ate O atoms (O5 and O5^i^) of the two symmetry-related, bidentately coordinated benzoate anions in the axial positions [symmetry code: (i) 1 − *x*, 2 − *y*, 1 − *z*] (Fig. 1 ▸, Table 1 ▸). On the other hand, the three carboxyl­ate O atoms (O2, O4 and O6) of the three bidentately coordinated benzoate anions around the Zn2 atom form a slightly distorted triangular planar arrangement, while the slightly distorted trigonal–pyramidal coordination sphere is completed by the pyridine N atom (N1) of the monodentately coordinated neutral pyridine-3-carbo­nitrile ligand in the axial position (Fig. 1 ▸, Table 1 ▸). The sum of the bond angles O2—Zn2—O4 [117.1 (2)°], O2—Zn2—O6 [111.1 (2)°] and O4—Zn2—O6 [127.4 (2)°] in the basal plane around the Zn2 atom is 355.6°. This confirms that the Zn2 atom deviates from the O2/O4/O6 basal plane; the deviation is 0.2390 (6) Å. The Zn1⋯Zn2 separation in the title trinuclear mol­ecule is 3.396 (2) Å and is comparable to the corresponding *M*—*M* distance (*M* is a metal) of 3.1845 (2) Å in the structurally related transition metal(II) complex [Zn_3_(benz)_6_(nia)_2_] (where benz = benzoate and nia = nicotinamide) (Zeleňák *et al.*, 2004 ▸). The volume of the polyhedron of atoms (Zn1/Zn2/O1–O6/C1/C8/C15) is calculated to be 15.62 (5) Å^3^.

The Zn1 and Zn2 atoms lie [0.7337 (1) and −0.1793 (6) Å], [1.0911 (1) and −0.2676 (6) Å] and [1.3428 (1) and 0.0520 (7) Å] above and/or below of the planar (O1/O2/C1), (O3/O4/C8) and (O5/O6/C15) carboxyl­ate groups, respect­ively. The (O1/O2/C1), (O3/O4/C8) and (O5/O6/C15) carboxyl­ate groups are twisted away from the attached benzene (*A*, C2—C7; *B*, C9—C14; *C*, C16—C21) rings by 6.4 (3), 26.5 (3) and 5.1 (3)°, respectively, while the benzene and pyridine (*D*, N1/C22—C26) rings are oriented at dihedral angles of *A*/*B* = 76.2 (2), *A*/*C* = 82.9 (2), *A*/*D* = 6.2 (2), *B*/*C* = 89.2 (2), *B*/*D* = 70.0 (2) and *C*/*D* = 83.0 (2)°.

## Supra­molecular features   

**Figure 2:**
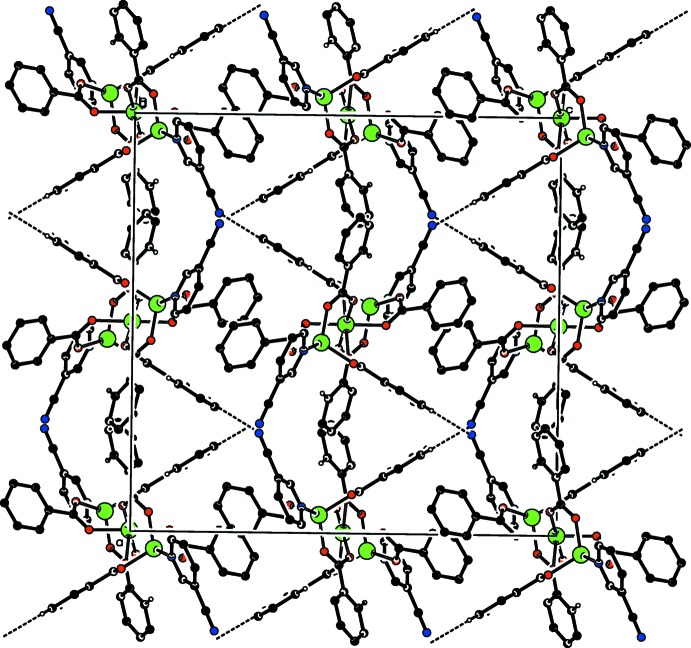
Part of the crystal structure. Weak C—H_Bnz_⋯N_Cpy_ (Bnz = benzoate and Cpy = pyridine-3-carbo­nitrile) hydrogen bonds are shown as dashed lines. H atoms not involved in these inter­actions have been omitted for clarity.

In the crystal, weak C—H_Bnz_⋯N_Cpy_ (Bnz = benzoate and Cpy = pyridine-3-carbo­nitrile) hydrogen bonds (Table 2 ▸) link the mol­ecules into a two-dimensional network parallel to (010) (Fig. 2 ▸). C—H⋯π and π–π inter­actions [between the benzene and pyridine rings of adjacent mol­ecules with an inter-centroid distance of 3.850 (4) Å] help to consolidate a three-dimensional architecture.

## Hirshfeld surface analysis   

Visualization and exploration of inter­molecular close contacts of a structure is invaluable, and this can be achieved using the Hirshfeld surface (HS) (Hirshfeld, 1977 ▸). HS analysis may be carried out to investigate the locations of atoms⋯atom short contacts with potential to form hydrogen bonds and π-stacking inter­actions.

**Figure 3:**
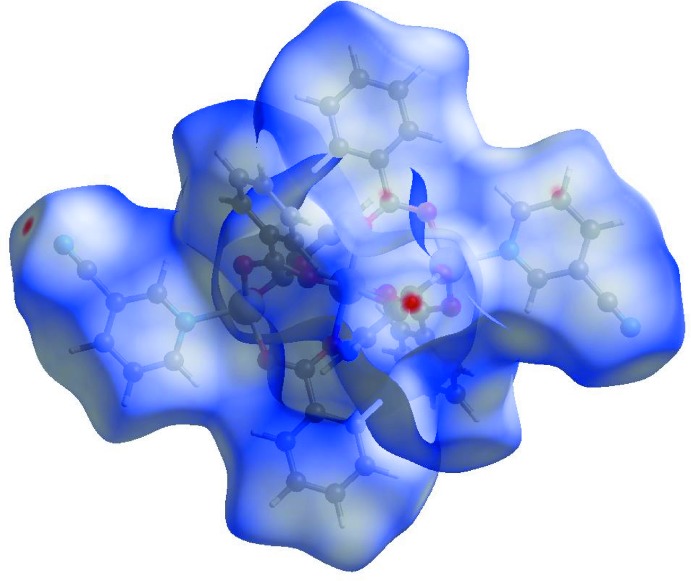
View of the three-dimensional Hirshfeld surface of the title complex plotted over *d*_norm_ in the range −0.0957 to 1.6461 a.u.

**Figure 4:**
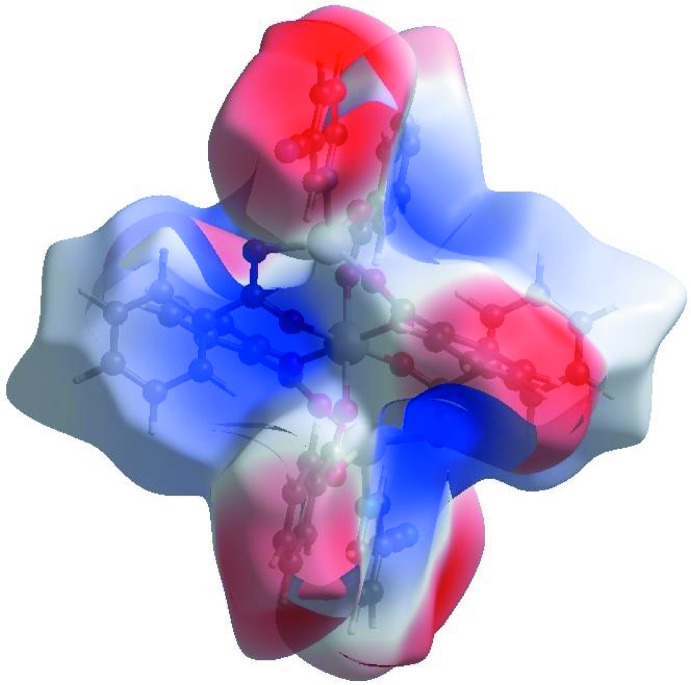
View of the three-dimensional Hirshfeld surface of the title complex plotted over electrostatic potential energy in the range −1.7824 to 9.8050 a.u. The hydrogen-bond donors and acceptors are viewed as blue and red regions around the atoms corresponding to positive and negative potentials, respectively.

**Figure 5:**
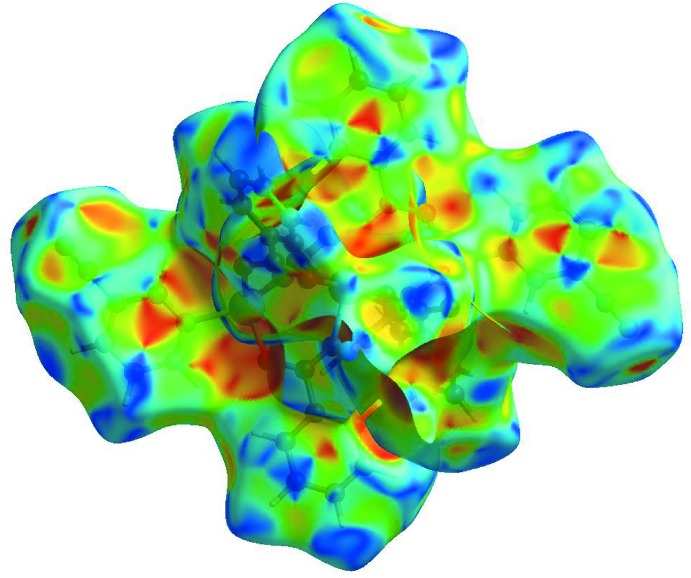
Hirshfeld surface of the title complex plotted over shape-index.

In the HS with *d*_norm_ (Fig. 3 ▸), the white surface indicates contacts with distances equal to the sum of van der Waals radii, and the red and blue colours indicate distances shorter (in close contact) or longer (distant contact) than the van der Waals radii, respectively. The bright-red spot appearing near Cpy-N2 indicates its role as the respective donor and/or acceptor in the dominant C—H⋯N hydrogen bond; it also appears as blue and/or red regions, respectively, corresponding to positive or negative potentials on the HS mapped over electrostatic potential (Fig. 4 ▸). The shape-index of the HS is a tool to visualize the π–π stacking by the presence of adjacent red and/or blue triangles; if there are no adjacent red and/or blue triangles, then there are no π–π inter­actions. Fig. 5 ▸ clearly suggests that there are π–π inter­actions in (I).

**Figure 6:**
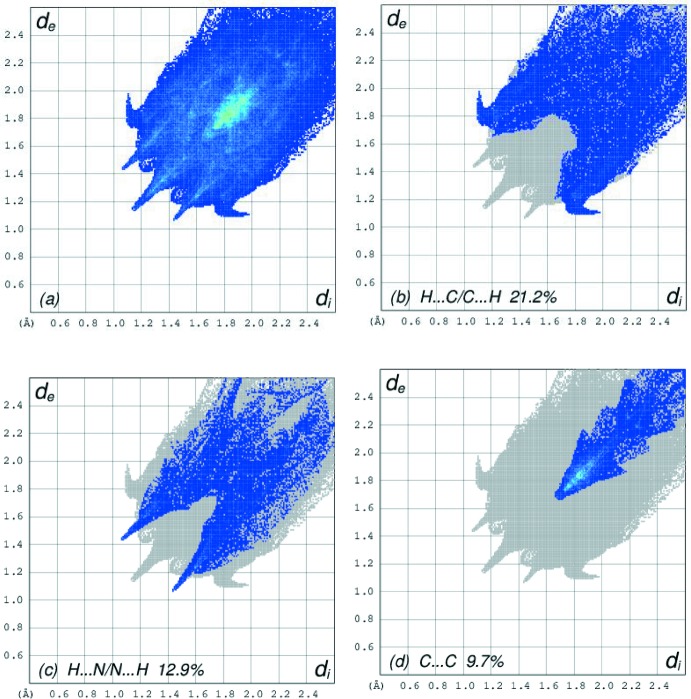
The selected two-dimensional fingerprint plots for the title complex, showing (*a*) all inter­actions, and delineated into (*b*) H⋯C/C⋯H, (*c*) H⋯N/N⋯H and (*d*) C⋯C inter­actions. The *d*_i_ and *d*_e_ values are the closest inter­nal and external distances (in Å) from given points on the Hirshfeld surface contacts.

The overall two-dimensional fingerprint plot, Fig. 6 ▸ *a*, and those delineated into H⋯C/C⋯H, H⋯N/N⋯H and C⋯C contacts are illustrated in Fig. 6 ▸ *b*–*d*, respectively, together with their relative contributions to the Hirshfeld surface. The widely scattered points of high density are due to the C—H⋯π inter­actions in the crystal, resulting in the fingerprint plot delineated into H⋯C/C⋯H contacts with 21.2% contribution to the HS, Fig. 6 ▸ *b*. In the fingerprint plot delin­eated into H⋯N / N⋯H contacts, the 12.9% contribution to the HS arises from the C—H⋯N hydrogen bonding and is viewed as a pair of spikes with the tip at *d*_e_ + *d*_i_ ∼2.6 Å in Fig. 6 ▸ *c*. Finally, the C⋯C contacts assigned to short inter­atomic C⋯C contacts and π–π stacking inter­actions with 9.7% contribution to the HS appear as an arrow-shaped distribution of points in Fig. 6 ▸ *d*, with the vertex at *d*_e_ = *d*_i_ ∼1.65 Å.

**Figure 7:**
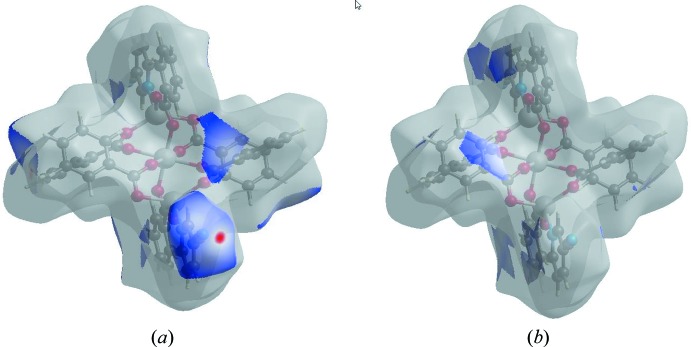
The Hirshfeld surface representations with the function *d*_norm_ plotted onto the surface for (*a*) H⋯N / N⋯H and (*b*) C⋯C inter­actions.

The Hirshfeld surface representations with the function *d*_norm_ plotted onto the surface are shown for the H⋯N/N⋯H and C⋯C inter­actions in Fig. 7*a* and *b* ▸, respectively.

The Hirshfeld surface analysis confirms the importance of H-atom contacts in establishing the packing. The crystal packing is dominated by van der Waals inter­actions and hydrogen bonding.

## Synthesis and crystallization   

The title compound was prepared by the reaction of ZnSO_4_·7H_2_O (1.44 g, 5 mmol) in H_2_O (25 ml) and pyridine-3-carbo­nitrile (1.04 g, 10 mmol) in water (25 ml) with sodium benzoate (1.44 g, 10 mmol) in water (100 ml) at room temperature. The mixture was filtered and set aside to crystallize at ambient temperature for several days, giving colourless single crystals (yield: 1.55 g, 82%).

## Refinement   

Crystal data, data collection and structure refinement details are summarized in Table 3 ▸. H atoms were positioned geom­etrically with C—H = 0.93 Å and constrained to ride on their parent atoms [*U*_iso_(H) = 1.2*U*_eq_(C)].

## Supplementary Material

Crystal structure: contains datablock(s) I, global. DOI: 10.1107/S2056989017016899/nk2240sup1.cif

Structure factors: contains datablock(s) I. DOI: 10.1107/S2056989017016899/nk2240Isup2.hkl

CCDC reference: 1587257

Additional supporting information:  crystallographic information; 3D view; checkCIF report

## Figures and Tables

**Table 1:** Selected bond lengths (Å)

Zn1—O1	2.039 (4)	Zn2—O4	1.963 (4)
Zn1—O3	2.117 (3)	Zn2—O6	2.034 (4)
Zn1—O5	2.099 (4)	Zn2—N1	2.091 (4)
Zn2—O2	1.948 (4)		

**Table 2:** Hydrogen-bond geometry (Å, °) *Cg* is the centroid of the C19–C14 ring.

*D*—H⋯*A*	*D*—H	H⋯*A*	*D*⋯*A*	*D*—H⋯*A*
C20—H20⋯N2^i^	0.93	2.63	3.448 (11)	147 (1)
C24—H24⋯*Cg* ^ii^	0.93	2.70	3.512 (6)	147

Symmetry codes: (i) 
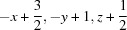
; (ii) 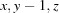.

**Table 3:** Experimental details

Crystal data	
Chemical formula	[Zn_3_(C_6_H_5_O_2_)_6_(C_6_H_4_N_2_)_2_]
*M*_r_	1130.99
Crystal system, space group	Orthorhombic, *P* *b* *c* *a*
Temperature (K)	296
*a*, *b*, *c* (Å)	21.7698 (4), 10.7768 (2), 22.2272 (4)
*V* (Å^3^)	5214.70 (17)
*Z*	4
Radiation type	Mo *K*α
μ (mm^−1^)	1.43
Crystal size (mm)	0.25 × 0.15 × 0.14

Data collection	
Diffractometer	Bruker APEXII CCD
Absorption correction	Multi-scan (*SADABS*; Bruker, 2012 ▸)
*T*_min_, *T*_max_	0.769, 0.805
No. of measured, independent and observed [*I* > 2σ(*I*)] reflections	57535, 5205, 4090
*R*_int_	0.044
(sin θ/λ)_max_ (Å^−1^)	0.624

Refinement	
*R*[*F* ^2^ > 2σ(*F* ^2^)], *wR*(*F* ^2^), *S*	0.061, 0.136, 1.25
No. of reflections	5205
No. of parameters	332
H-atom treatment	H-atom parameters constrained
Δρ_max_, Δρ_min_ (e Å^−3^)	0.51, −0.53

Computer programs: *APEX2* and *SAINT* (Bruker, 2012 ▸), *SHELXS97* and *SHELXL97* (Sheldrick, 2008 ▸), *ORTEP-3 for Windows* and *WinGX* (Farrugia, 2012 ▸) and *PLATON* (Spek, 2009 ▸).

## supplementary crystallographic information

### Crystal data

**Table:**

[Zn_3_(C_6_H_5_O_2_)_6_(C_6_H_4_N_2_)_2_]	*F*(000) = 2304
*M**_r_* = 1130.99	*D*_x_ = 1.441 Mg m^−^^3^
Orthorhombic, *P**b**c**a*	Mo *K*α radiation, λ = 0.71073 Å
Hall symbol: -P 2ac 2ab	Cell parameters from 9334 reflections
*a* = 21.7698 (4) Å	θ = 3.2–26.4°
*b* = 10.7768 (2) Å	µ = 1.43 mm^−^^1^
*c* = 22.2272 (4) Å	*T* = 296 K
*V* = 5214.70 (17) Å^3^	Prism, colourless
*Z* = 4	0.25 × 0.15 × 0.14 mm

### Data collection

**Table:**

Bruker APEXII CCD diffractometer	5205 independent reflections
Radiation source: fine-focus sealed tube	4090 reflections with *I* > 2σ(*I*)
Graphite monochromator	*R*_int_ = 0.044
φ and ω scans	θ_max_ = 26.3°, θ_min_ = 2.8°
Absorption correction: multi-scan (SADABS; Bruker, 2012)	*h* = −27→27
*T*_min_ = 0.769, *T*_max_ = 0.805	*k* = −12→9
57535 measured reflections	*l* = −27→27

### Refinement

**Table:**

Refinement on *F*^2^	Secondary atom site location: difference Fourier map
Least-squares matrix: full	Hydrogen site location: inferred from neighbouring sites
*R*[*F*^2^ > 2σ(*F*^2^)] = 0.061	H-atom parameters constrained
*wR*(*F*^2^) = 0.136	*w* = 1/[σ^2^(*F*_o_^2^) + (0.*P*)^2^ + 23.0318*P*] where *P* = (*F*_o_^2^ + 2*F*_c_^2^)/3
*S* = 1.25	(Δ/σ)_max_ = 0.001
5205 reflections	Δρ_max_ = 0.51 e Å^−^^3^
332 parameters	Δρ_min_ = −0.53 e Å^−^^3^
0 restraints	Extinction correction: *SHELXL97* (Sheldrick, 2008), Fc^*^=kFc[1+0.001xFc^2^λ^3^/sin(2θ)]^-1/4^
Primary atom site location: structure-invariant direct methods	Extinction coefficient: 0.0033 (2)

### Special details

**Table:**

Geometry. All esds (except the esd in the dihedral angle between two l.s. planes) are estimated using the full covariance matrix. The cell esds are taken into account individually in the estimation of esds in distances, angles and torsion angles; correlations between esds in cell parameters are only used when they are defined by crystal symmetry. An approximate (isotropic) treatment of cell esds is used for estimating esds involving l.s. planes.
Refinement. Refinement of F^2^ against ALL reflections. The weighted R-factor wR and goodness of fit S are based on F^2^, conventional R-factors R are based on F, with F set to zero for negative F^2^. The threshold expression of F^2^ > 2sigma(F^2^) is used only for calculating R-factors(gt) etc. and is not relevant to the choice of reflections for refinement. R-factors based on F^2^ are statistically about twice as large as those based on F, and R- factors based on ALL data will be even larger.

### Fractional atomic coordinates and isotropic or equivalent isotropic displacement parameters (Å^2^)

**Table:**

	*x*	*y*	*z*	*U*_iso_*/*U*_eq_	
Zn1	0.5000	1.0000	0.5000	0.0328 (2)	
Zn2	0.54302 (3)	0.72121 (5)	0.44260 (3)	0.03771 (19)	
O1	0.4419 (2)	0.8554 (4)	0.51623 (18)	0.0620 (12)	
O2	0.45578 (16)	0.6924 (3)	0.45634 (16)	0.0465 (9)	
O3	0.49893 (17)	0.9704 (4)	0.40584 (14)	0.0465 (9)	
O4	0.56537 (18)	0.8269 (3)	0.37406 (16)	0.0488 (9)	
O5	0.57926 (19)	0.8901 (4)	0.50701 (17)	0.0606 (12)	
O6	0.5925 (2)	0.6912 (4)	0.51883 (19)	0.0621 (11)	
N1	0.5624 (2)	0.5518 (4)	0.40092 (19)	0.0405 (10)	
N2	0.7379 (3)	0.3920 (7)	0.2993 (3)	0.097 (2)	
C1	0.4237 (2)	0.7599 (5)	0.4913 (2)	0.0385 (11)	
C2	0.3595 (2)	0.7168 (5)	0.5033 (2)	0.0452 (12)	
C3	0.3211 (3)	0.7890 (7)	0.5383 (3)	0.074 (2)	
H3	0.3343	0.8653	0.5529	0.089*	
C4	0.2615 (4)	0.7454 (11)	0.5515 (5)	0.112 (4)	
H4	0.2352	0.7932	0.5749	0.135*	
C5	0.2423 (4)	0.6337 (12)	0.5300 (5)	0.115 (4)	
H5	0.2029	0.6057	0.5386	0.137*	
C6	0.2798 (4)	0.5641 (9)	0.4966 (4)	0.100 (3)	
H6	0.2663	0.4875	0.4826	0.120*	
C7	0.3387 (3)	0.6039 (7)	0.4822 (3)	0.0672 (18)	
H7	0.3640	0.5545	0.4585	0.081*	
C8	0.5356 (2)	0.9277 (5)	0.3681 (2)	0.0383 (11)	
C9	0.5447 (3)	0.9980 (5)	0.3106 (2)	0.0414 (12)	
C10	0.5983 (3)	0.9883 (6)	0.2789 (3)	0.0588 (16)	
H10	0.6289	0.9348	0.2923	0.071*	
C11	0.6075 (4)	1.0582 (7)	0.2266 (3)	0.078 (2)	
H11	0.6445	1.0533	0.2058	0.094*	
C12	0.5609 (4)	1.1346 (7)	0.2062 (3)	0.077 (2)	
H12	0.5664	1.1804	0.1712	0.092*	
C13	0.5067 (4)	1.1432 (6)	0.2372 (3)	0.0680 (19)	
H13	0.4755	1.1946	0.2231	0.082*	
C14	0.4982 (3)	1.0756 (5)	0.2896 (2)	0.0516 (14)	
H14	0.4615	1.0820	0.3107	0.062*	
C15	0.5997 (2)	0.8003 (6)	0.5365 (2)	0.0447 (13)	
C16	0.6358 (2)	0.8249 (5)	0.5925 (2)	0.0442 (12)	
C17	0.6485 (3)	0.9441 (6)	0.6100 (3)	0.0621 (16)	
H17	0.6345	1.0107	0.5871	0.075*	
C18	0.6823 (4)	0.9651 (8)	0.6621 (4)	0.089 (2)	
H18	0.6914	1.0458	0.6739	0.106*	
C19	0.7022 (4)	0.8669 (10)	0.6959 (4)	0.098 (3)	
H19	0.7250	0.8815	0.7306	0.118*	
C20	0.6892 (4)	0.7477 (10)	0.6794 (4)	0.102 (3)	
H20	0.7028	0.6814	0.7026	0.122*	
C21	0.6555 (3)	0.7270 (7)	0.6276 (3)	0.0717 (19)	
H21	0.6459	0.6461	0.6163	0.086*	
C22	0.6160 (2)	0.5327 (5)	0.3742 (2)	0.0457 (13)	
H22	0.6449	0.5962	0.3741	0.055*	
C23	0.6306 (3)	0.4213 (5)	0.3463 (2)	0.0478 (13)	
C24	0.5872 (3)	0.3277 (6)	0.3463 (3)	0.0590 (16)	
H24	0.5956	0.2521	0.3278	0.071*	
C25	0.5316 (3)	0.3474 (5)	0.3739 (3)	0.0573 (16)	
H25	0.5018	0.2857	0.3742	0.069*	
C26	0.5208 (3)	0.4603 (5)	0.4012 (2)	0.0465 (13)	
H26	0.4834	0.4732	0.4204	0.056*	
C27	0.6905 (3)	0.4056 (7)	0.3197 (3)	0.0641 (17)	

### Atomic displacement parameters (Å^2^)

**Table:**

	*U*^11^	*U*^22^	*U*^33^	*U*^12^	*U*^13^	*U*^23^
Zn1	0.0425 (4)	0.0234 (4)	0.0325 (4)	0.0037 (3)	−0.0005 (3)	−0.0020 (3)
Zn2	0.0439 (3)	0.0301 (3)	0.0391 (3)	0.0032 (2)	−0.0002 (3)	−0.0042 (2)
O1	0.082 (3)	0.044 (2)	0.060 (2)	−0.025 (2)	0.026 (2)	−0.0145 (19)
O2	0.0406 (19)	0.044 (2)	0.055 (2)	0.0035 (16)	0.0040 (17)	−0.0146 (17)
O3	0.050 (2)	0.060 (2)	0.0294 (17)	0.0131 (19)	0.0000 (16)	−0.0042 (16)
O4	0.067 (2)	0.035 (2)	0.044 (2)	0.0047 (18)	0.0064 (18)	−0.0003 (16)
O5	0.059 (3)	0.079 (3)	0.044 (2)	0.034 (2)	−0.0014 (19)	0.007 (2)
O6	0.074 (3)	0.057 (3)	0.056 (2)	−0.001 (2)	−0.015 (2)	−0.008 (2)
N1	0.046 (2)	0.033 (2)	0.043 (2)	0.0014 (19)	−0.0009 (19)	−0.0049 (18)
N2	0.077 (4)	0.122 (6)	0.091 (5)	0.026 (4)	0.035 (4)	−0.009 (4)
C1	0.043 (3)	0.032 (3)	0.041 (3)	−0.001 (2)	−0.002 (2)	0.005 (2)
C2	0.035 (3)	0.047 (3)	0.054 (3)	0.006 (2)	−0.002 (2)	0.012 (3)
C3	0.057 (4)	0.068 (5)	0.098 (5)	0.021 (3)	0.023 (4)	0.023 (4)
C4	0.059 (5)	0.129 (8)	0.149 (9)	0.039 (6)	0.038 (6)	0.048 (8)
C5	0.031 (4)	0.171 (11)	0.141 (9)	−0.007 (5)	−0.008 (5)	0.075 (9)
C6	0.060 (5)	0.121 (8)	0.120 (7)	−0.050 (5)	−0.019 (5)	0.037 (6)
C7	0.062 (4)	0.068 (4)	0.071 (4)	−0.018 (3)	−0.010 (3)	0.009 (4)
C8	0.048 (3)	0.034 (3)	0.033 (2)	−0.003 (2)	−0.007 (2)	−0.005 (2)
C9	0.057 (3)	0.037 (3)	0.030 (2)	−0.007 (3)	−0.002 (2)	−0.007 (2)
C10	0.070 (4)	0.059 (4)	0.048 (3)	−0.002 (3)	0.013 (3)	0.001 (3)
C11	0.097 (6)	0.084 (5)	0.055 (4)	−0.012 (5)	0.021 (4)	0.004 (4)
C12	0.124 (7)	0.067 (5)	0.039 (3)	−0.016 (5)	−0.001 (4)	0.010 (3)
C13	0.095 (5)	0.063 (4)	0.046 (4)	−0.004 (4)	−0.019 (4)	0.006 (3)
C14	0.065 (4)	0.049 (3)	0.041 (3)	−0.002 (3)	−0.008 (3)	−0.002 (2)
C15	0.033 (3)	0.061 (4)	0.039 (3)	0.006 (2)	0.001 (2)	−0.004 (3)
C16	0.042 (3)	0.052 (3)	0.038 (3)	0.005 (2)	−0.005 (2)	0.001 (2)
C17	0.059 (4)	0.060 (4)	0.067 (4)	0.000 (3)	−0.008 (3)	−0.005 (3)
C18	0.097 (6)	0.090 (6)	0.079 (5)	−0.019 (5)	−0.018 (5)	−0.025 (5)
C19	0.088 (6)	0.140 (9)	0.066 (5)	−0.005 (6)	−0.040 (4)	−0.016 (5)
C20	0.117 (7)	0.112 (7)	0.076 (5)	0.026 (6)	−0.048 (5)	0.006 (5)
C21	0.086 (5)	0.067 (4)	0.062 (4)	0.009 (4)	−0.030 (4)	0.003 (3)
C22	0.047 (3)	0.044 (3)	0.046 (3)	−0.003 (2)	−0.004 (2)	−0.005 (2)
C23	0.052 (3)	0.051 (3)	0.041 (3)	0.012 (3)	0.007 (2)	−0.008 (2)
C24	0.074 (4)	0.041 (3)	0.062 (4)	0.007 (3)	0.001 (3)	−0.015 (3)
C25	0.062 (4)	0.036 (3)	0.074 (4)	−0.004 (3)	0.003 (3)	−0.013 (3)
C26	0.050 (3)	0.040 (3)	0.049 (3)	0.006 (2)	0.006 (2)	0.001 (2)
C27	0.068 (4)	0.071 (4)	0.054 (4)	0.018 (3)	0.014 (3)	−0.006 (3)

### Geometric parameters (Å, º)

**Table:**

Zn1—O1	2.039 (4)	C8—C9	1.500 (7)
Zn1—O1^i^	2.039 (4)	C9—C10	1.367 (8)
Zn1—O3	2.117 (3)	C9—C14	1.393 (8)
Zn1—O3^i^	2.117 (3)	C10—C11	1.400 (9)
Zn1—O5	2.099 (4)	C10—H10	0.9300
Zn1—O5^i^	2.099 (4)	C11—C12	1.382 (10)
Zn2—O2	1.948 (4)	C11—H11	0.9300
Zn2—O4	1.963 (4)	C12—C13	1.370 (10)
Zn2—O5	2.446 (5)	C12—H12	0.9300
Zn2—O6	2.034 (4)	C13—C14	1.385 (8)
Zn2—N1	2.091 (4)	C13—H13	0.9300
Zn2—C15	2.571 (5)	C14—H14	0.9300
O1—C1	1.235 (6)	C15—C16	1.494 (7)
O2—C1	1.274 (6)	C16—C17	1.370 (8)
O3—C8	1.245 (6)	C16—C21	1.381 (8)
O4—C8	1.272 (6)	C17—C18	1.391 (9)
O5—C15	1.251 (7)	C17—H17	0.9300
O6—C15	1.250 (7)	C18—C19	1.369 (12)
N1—C22	1.326 (7)	C18—H18	0.9300
N1—C26	1.339 (7)	C19—C20	1.366 (12)
N2—C27	1.138 (8)	C19—H19	0.9300
C1—C2	1.496 (7)	C20—C21	1.384 (9)
C2—C7	1.381 (8)	C20—H20	0.9300
C2—C3	1.383 (8)	C21—H21	0.9300
C3—C4	1.411 (11)	C22—C23	1.387 (7)
C3—H3	0.9300	C22—H22	0.9300
C4—C5	1.361 (14)	C23—C24	1.382 (8)
C4—H4	0.9300	C23—C27	1.441 (8)
C5—C6	1.335 (14)	C24—C25	1.373 (8)
C5—H5	0.9300	C24—H24	0.9300
C6—C7	1.388 (9)	C25—C26	1.379 (7)
C6—H6	0.9300	C25—H25	0.9300
C7—H7	0.9300	C26—H26	0.9300
			
O1^i^—Zn1—O1	180.000 (1)	O3—C8—O4	124.9 (5)
O1^i^—Zn1—O5	86.25 (19)	O3—C8—C9	118.2 (5)
O1—Zn1—O5	93.75 (19)	O4—C8—C9	116.9 (5)
O1^i^—Zn1—O5^i^	93.75 (19)	C10—C9—C14	119.6 (5)
O1—Zn1—O5^i^	86.25 (19)	C10—C9—C8	120.9 (5)
O5—Zn1—O5^i^	180.000 (1)	C14—C9—C8	119.5 (5)
O1^i^—Zn1—O3	86.97 (16)	C9—C10—C11	120.5 (7)
O1—Zn1—O3	93.03 (16)	C9—C10—H10	119.7
O5—Zn1—O3	89.85 (14)	C11—C10—H10	119.7
O5^i^—Zn1—O3	90.15 (14)	C12—C11—C10	119.2 (7)
O1^i^—Zn1—O3^i^	93.03 (16)	C12—C11—H11	120.4
O1—Zn1—O3^i^	86.97 (16)	C10—C11—H11	120.4
O5—Zn1—O3^i^	90.15 (14)	C13—C12—C11	120.5 (6)
O5^i^—Zn1—O3^i^	89.85 (14)	C13—C12—H12	119.8
O3—Zn1—O3^i^	180.000 (1)	C11—C12—H12	119.8
O2—Zn2—O4	117.11 (16)	C12—C13—C14	120.1 (7)
O2—Zn2—O6	111.11 (17)	C12—C13—H13	119.9
O4—Zn2—O6	127.42 (18)	C14—C13—H13	119.9
O2—Zn2—N1	97.29 (16)	C13—C14—C9	120.0 (6)
O4—Zn2—N1	96.46 (16)	C13—C14—H14	120.0
O6—Zn2—N1	97.08 (17)	C9—C14—H14	120.0
O2—Zn2—O5	109.96 (14)	O6—C15—O5	121.2 (5)
O4—Zn2—O5	86.72 (15)	O6—C15—C16	119.7 (5)
O6—Zn2—O5	57.31 (15)	O5—C15—C16	119.1 (5)
N1—Zn2—O5	147.56 (15)	O6—C15—Zn2	51.1 (3)
O2—Zn2—C15	113.20 (16)	O5—C15—Zn2	70.1 (3)
O4—Zn2—C15	108.58 (17)	C16—C15—Zn2	170.7 (4)
O6—Zn2—C15	28.58 (17)	C17—C16—C21	119.6 (6)
N1—Zn2—C15	123.54 (17)	C17—C16—C15	120.6 (5)
O5—Zn2—C15	28.74 (15)	C21—C16—C15	119.8 (5)
C1—O1—Zn1	139.2 (4)	C16—C17—C18	119.7 (7)
C1—O2—Zn2	122.6 (3)	C16—C17—H17	120.1
C8—O3—Zn1	135.6 (3)	C18—C17—H17	120.1
C8—O4—Zn2	116.7 (3)	C19—C18—C17	120.0 (8)
C15—O5—Zn1	140.2 (4)	C19—C18—H18	120.0
C15—O5—Zn2	81.2 (3)	C17—C18—H18	120.0
Zn1—O5—Zn2	96.39 (16)	C20—C19—C18	120.9 (7)
C15—O6—Zn2	100.3 (4)	C20—C19—H19	119.6
C22—N1—C26	118.9 (5)	C18—C19—H19	119.6
C22—N1—Zn2	120.8 (4)	C19—C20—C21	119.1 (8)
C26—N1—Zn2	120.3 (4)	C19—C20—H20	120.4
O1—C1—O2	125.0 (5)	C21—C20—H20	120.4
O1—C1—C2	118.7 (5)	C16—C21—C20	120.8 (7)
O2—C1—C2	116.3 (5)	C16—C21—H21	119.6
C7—C2—C3	119.2 (6)	C20—C21—H21	119.6
C7—C2—C1	121.3 (5)	N1—C22—C23	122.4 (5)
C3—C2—C1	119.4 (6)	N1—C22—H22	118.8
C2—C3—C4	119.1 (8)	C23—C22—H22	118.8
C2—C3—H3	120.5	C24—C23—C22	118.4 (5)
C4—C3—H3	120.5	C24—C23—C27	122.1 (5)
C5—C4—C3	120.2 (9)	C22—C23—C27	119.5 (6)
C5—C4—H4	119.9	C25—C24—C23	119.3 (5)
C3—C4—H4	119.9	C25—C24—H24	120.4
C6—C5—C4	120.4 (8)	C23—C24—H24	120.4
C6—C5—H5	119.8	C24—C25—C26	118.9 (6)
C4—C5—H5	119.8	C24—C25—H25	120.6
C5—C6—C7	121.3 (9)	C26—C25—H25	120.6
C5—C6—H6	119.4	N1—C26—C25	122.2 (5)
C7—C6—H6	119.4	N1—C26—H26	118.9
C2—C7—C6	119.8 (8)	C25—C26—H26	118.9
C2—C7—H7	120.1	N2—C27—C23	179.1 (8)
C6—C7—H7	120.1		
			
O5—Zn1—O1—C1	−70.0 (6)	C2—C3—C4—C5	0.1 (13)
O5^i^—Zn1—O1—C1	110.0 (6)	C3—C4—C5—C6	0.4 (15)
O3—Zn1—O1—C1	20.0 (6)	C4—C5—C6—C7	−0.8 (15)
O3^i^—Zn1—O1—C1	−160.0 (6)	C3—C2—C7—C6	−0.3 (10)
O4—Zn2—O2—C1	−92.5 (4)	C1—C2—C7—C6	176.9 (6)
O6—Zn2—O2—C1	65.8 (4)	C5—C6—C7—C2	0.7 (12)
N1—Zn2—O2—C1	166.4 (4)	Zn1—O3—C8—O4	47.4 (8)
O5—Zn2—O2—C1	4.2 (4)	Zn1—O3—C8—C9	−133.9 (4)
C15—Zn2—O2—C1	35.0 (4)	Zn2—O4—C8—O3	8.8 (7)
O1^i^—Zn1—O3—C8	73.7 (5)	Zn2—O4—C8—C9	−169.9 (3)
O1—Zn1—O3—C8	−106.3 (5)	O3—C8—C9—C10	153.9 (5)
O5—Zn1—O3—C8	−12.5 (5)	O4—C8—C9—C10	−27.4 (7)
O5^i^—Zn1—O3—C8	167.5 (5)	O3—C8—C9—C14	−25.2 (7)
O2—Zn2—O4—C8	46.3 (4)	O4—C8—C9—C14	153.6 (5)
O6—Zn2—O4—C8	−108.0 (4)	C14—C9—C10—C11	1.8 (9)
N1—Zn2—O4—C8	147.9 (4)	C8—C9—C10—C11	−177.3 (6)
O5—Zn2—O4—C8	−64.5 (4)	C9—C10—C11—C12	−1.9 (10)
C15—Zn2—O4—C8	−83.4 (4)	C10—C11—C12—C13	0.9 (11)
O1^i^—Zn1—O5—C15	143.5 (6)	C11—C12—C13—C14	0.2 (11)
O1—Zn1—O5—C15	−36.5 (6)	C12—C13—C14—C9	−0.4 (9)
O3—Zn1—O5—C15	−129.6 (6)	C10—C9—C14—C13	−0.6 (8)
O3^i^—Zn1—O5—C15	50.4 (6)	C8—C9—C14—C13	178.5 (5)
O1^i^—Zn1—O5—Zn2	−132.68 (16)	Zn2—O6—C15—O5	−1.5 (6)
O1—Zn1—O5—Zn2	47.32 (16)	Zn2—O6—C15—C16	−178.8 (4)
O3—Zn1—O5—Zn2	−45.71 (15)	Zn1—O5—C15—O6	90.6 (7)
O3^i^—Zn1—O5—Zn2	134.29 (15)	Zn2—O5—C15—O6	1.2 (5)
O2—Zn2—O5—C15	102.1 (3)	Zn1—O5—C15—C16	−92.0 (7)
O4—Zn2—O5—C15	−140.2 (3)	Zn2—O5—C15—C16	178.6 (5)
O6—Zn2—O5—C15	−0.8 (3)	Zn1—O5—C15—Zn2	89.4 (6)
N1—Zn2—O5—C15	−43.3 (5)	O2—Zn2—C15—O6	91.9 (4)
O2—Zn2—O5—Zn1	−37.85 (19)	O4—Zn2—C15—O6	−136.3 (4)
O4—Zn2—O5—Zn1	79.85 (17)	N1—Zn2—C15—O6	−24.9 (4)
O6—Zn2—O5—Zn1	−140.7 (2)	O5—Zn2—C15—O6	−178.7 (6)
N1—Zn2—O5—Zn1	176.7 (2)	O2—Zn2—C15—O5	−89.5 (3)
C15—Zn2—O5—Zn1	−139.9 (4)	O4—Zn2—C15—O5	42.4 (3)
O2—Zn2—O6—C15	−100.0 (4)	O6—Zn2—C15—O5	178.7 (6)
O4—Zn2—O6—C15	55.6 (4)	N1—Zn2—C15—O5	153.8 (3)
N1—Zn2—O6—C15	159.3 (4)	O6—C15—C16—C17	174.7 (6)
O5—Zn2—O6—C15	0.8 (3)	O5—C15—C16—C17	−2.7 (8)
O2—Zn2—N1—C22	169.8 (4)	O6—C15—C16—C21	−6.6 (8)
O4—Zn2—N1—C22	51.3 (4)	O5—C15—C16—C21	176.0 (6)
O6—Zn2—N1—C22	−77.8 (4)	C21—C16—C17—C18	1.6 (10)
O5—Zn2—N1—C22	−42.7 (5)	C15—C16—C17—C18	−179.7 (6)
C15—Zn2—N1—C22	−66.1 (4)	C16—C17—C18—C19	−0.6 (12)
O2—Zn2—N1—C26	−10.7 (4)	C17—C18—C19—C20	−0.3 (14)
O4—Zn2—N1—C26	−129.2 (4)	C18—C19—C20—C21	0.2 (15)
O6—Zn2—N1—C26	101.8 (4)	C17—C16—C21—C20	−1.7 (11)
O5—Zn2—N1—C26	136.8 (4)	C15—C16—C21—C20	179.6 (7)
C15—Zn2—N1—C26	113.5 (4)	C19—C20—C21—C16	0.8 (13)
Zn1—O1—C1—O2	33.4 (9)	C26—N1—C22—C23	0.2 (8)
Zn1—O1—C1—C2	−147.8 (5)	Zn2—N1—C22—C23	179.7 (4)
Zn2—O2—C1—O1	6.3 (7)	N1—C22—C23—C24	0.4 (9)
Zn2—O2—C1—C2	−172.5 (3)	N1—C22—C23—C27	−178.1 (5)
O1—C1—C2—C7	−172.7 (5)	C22—C23—C24—C25	−0.3 (9)
O2—C1—C2—C7	6.2 (7)	C27—C23—C24—C25	178.1 (6)
O1—C1—C2—C3	4.5 (8)	C23—C24—C25—C26	−0.3 (10)
O2—C1—C2—C3	−176.6 (5)	C22—N1—C26—C25	−0.9 (8)
C7—C2—C3—C4	−0.1 (10)	Zn2—N1—C26—C25	179.6 (4)
C1—C2—C3—C4	−177.4 (6)	C24—C25—C26—N1	0.9 (9)

Symmetry code: (i) −*x*+1, −*y*+2, −*z*+1.

### Hydrogen-bond geometry (Å, º)

Cg is the centroid of the C19–C14 ring.

**Table:**

*D*—H···*A*	*D*—H	H···*A*	*D*···*A*	*D*—H···*A*
C20—H20···N2^ii^	0.93	2.63	3.448 (11)	147 (1)
C24—H24···*Cg*^iii^	0.93	2.70	3.512 (6)	147

Symmetry codes: (ii) −*x*+3/2, −*y*+1, *z*+1/2; (iii) *x*, *y*−1, *z*.
